# Mortality of CC398 *Staphylococcus aureus* bloodstream infections among hospitalised patients in a Swiss university hospital: a retrospective cohort study

**DOI:** 10.1007/s10096-026-05493-6

**Published:** 2026-04-07

**Authors:** Davide Bosetti, Rebecca Grant, Etienne Chalot, Mohamed Abbas, Niccolò Buetti, Patrice François, Stephan Harbarth

**Affiliations:** 1 Infection Control Programme and WHO Collaborating Centre, Faculty of Medicine, Geneva University Hospitals, Rue Gabrielle-Perret-Gentil 4, Geneva, 1205 Switzerland; 2 Division of Infectious Diseases, Geneva University Hospitals, Rue Gabrielle-Perret-Gentil 4, Geneva, 1205 Switzerland; 3 Infection Antimicrobials Modelling Evolution, INSERM, University of Paris, Rue d‘Oradour-sur-Glane 10, Paris, 75015 France; 4 Bacteriology Laboratory, Division of Laboratory Medicine, Geneva University Hospitals, Rue Gabrielle-Perret-Gentil 4, Geneva, 1205 Switzerland

**Keywords:** *Staphylococcus aureus*, Methicillin-susceptible *Staphylococcus aureus*, Clonal complex 398, Epidemiology

## Abstract

Methicillin-susceptible *Staphylococcus aureus* (MSSA) clonal complex 398 (CC398) bloodstream infections (BSI) have been increasingly reported and were previously suggested to be associated with increased mortality. We conducted a retrospective cohort study of hospitalised patients with MSSA BSI at Geneva University Hospitals between 2021 and 2023. Among 294 MSSA BSI, 57 (19%) were CC398 and 185 (63%) were healthcare-associated. Crude 90-day all-cause mortality was similar in both groups (19% CC398, 22% non-CC398, *p* = 0.79). In adjusted analyses, there was no difference in the all-cause mortality hazard between CC398 and non-CC398 patients (aHR 30-day mortality: 0.87, 95%CI:0.41–1.83; aHR 90-day mortality 1.17, 95%CI:0.61–2.25).

**Supplementary Information:**

The online version contains supplementary material available at https://doi.org/10.1007/s10096-026-05493-6.

## Introduction

*Staphylococcus aureus* bloodstream infections (BSI) remain a major cause of morbidity and mortality worldwide. Recent estimates of 30-day all-cause mortality from S. aureus BSI range between 16 and 20%, depending on host factors, source of infection, and timely source control [1]. In Western Europe, the burden of *S. aureus* BSI remains substantial, with methicillin-susceptible *S. aureus* (MSSA) now responsible for a large proportion of *S. aureus* BSI, while methicillin-resistant *S. aureus* (MRSA) has substantially declined [2]. Similarly in Switzerland, the incidence of MSSA BSI has increased substantially (+ 37% from 17.8 to 24.4 per 100,000 inhabitants) between 2008 and 2021, while the incidence of MRSA BSI during the same period has declined (from 1.9 to 1.2 per 100,000 inhabitants) [3].

Within MSSA strains, clonal complex 398 (CC398) has emerged and expanded in prevalence in several regions. Phylogenomic analyses suggest that CC398 originated as a human-associated lineage, with livestock-associated and human clades subsequently diverging from a common ancestor [4–6]. The human-adapted clade, most frequently reported in France, Spain and China, is thought to have developed distinct transmission and virulence features [7]. Further, an initial case-control study of patients with *S. aureus* BSI suggested that MSSA BSI due to CC398 may be associated with increased healthcare acquisition and mortality [8]. To confirm these findings and given the limited evidence as to the clinical impact of CC398, we conducted a retrospective cohort study comparing mortality of CC398 and non-CC398 MSSA BSI among hospitalised patients between 2021 and 2023.

## Methods

### Study design and inclusion criteria

We conducted a retrospective, single-centre observational cohort study at Geneva University Hospitals (HUG), a 2,100-bed academic tertiary-care institution in Switzerland, in which the proportion of CC398 among MSSA BSI has increased from < 5% in 2009 to > 20% in 2023 (data not shown). We included all hospitalised patients who had a MSSA positive blood culture between 1 January 2021 and 31 December 2023. For patients with multiple MSSA BSI during the study period, only the first episode was considered. Patients excluded from this analysis were those with a stored MSSA isolate that was not retrievable or analysable; or those who refused the hospital’s general consent of the reuse of health data and samples for research purposes (Supplementary Fig. 1).

### Microbiological and molecular characterization

As routine procedure, MSSA BSI isolates are stored by the Bacteriology Laboratory in a biobank. All isolates included in this analysis were retrieved, subcultured on blood agar plates, and genotyped using a previously described MLVA assay [9] with slight modifications to distinguish CC398 isolates from non-CC398 isolates (see Supplementary Material).

### Data collection

Demographic, clinical and hospitalisation-related data were extracted from patients’ electronic health records (EHR), including: birth year; sex; weighted Charlson comorbidity score [10]; catheter insertion prior to current hospitalisation; concurrent influenza or SARS-CoV-2 infection, defined as a positive test result within 7 and 14 days of MSSA positive blood culture collection, respectively; dates of current and prior hospital admission and blood culture collection; intermediate (IMCU) or intensive care unit (ICU) admission; and clinical outcome (date of discharge, date of death). All-cause mortality included both in-hospital deaths recorded in EHR, as well as out-of-hospital deaths which are registered in the cantonal death registry.

### Outcomes

The primary outcome was all-cause mortality within 90 days following a positive MSSA blood culture. Secondary outcomes included all-cause mortality within 30 days, admission to the IMCU or ICU following a positive MSSA blood culture and length of hospital stay (LOS) after MSSA BSI onset.

### Statistical analyses

We described the characteristics and clinical outcomes of CC398 and non-CC398 MSSA BSI included in the analysis. Continuous variables were expressed as median and interquartile range (IQR) and categorical variables as counts and percentages. Differences between groups were assessed using Wilcoxon rank sum tests for continuous variables and chi-squared or Fischer’s exact tests for categorical variables, as appropriate.

We plotted cumulative incidence functions for 90-day all-cause mortality. Patients who remained hospitalised for more than 90 days after the date of blood culture collection were censored.

We stratified results by community-acquired versus healthcare-associated BSI. Similar to the conservative definition used by Friedman ND et al. [11], and Bouiller K et al. [8], BSI were considered to be healthcare-associated if they met any of the following criteria: (i) prior hospitalization at HUG for more than 48 h within the previous 90 days, or (ii) positive blood culture collected more than 48 h after hospital admission, or (iii) presence of a central venous catheter, peripherally inserted central catheter, or dialysis access via intravascular device at hospital admission, or (iv) history of hemodialysis, or (iv) residence in a long-term care facility. All other BSI were considered to be community-acquired. We compared 90-day cumulative incidence of all-cause mortality between CC398 and non-CC398 for community-acquired and healthcare-associated MSSA BSI using weighted log-rank test (rho = 1) to account for potential non-proportional hazards.

In adjusted analyses a multivariable Cox proportional hazards model was used including clinically relevant covariates: CC398 versus non-CC398, (covariate of interest), age, sex, weighted Charlson comorbidity score (derived from ICD-10 codes), and BSI classification (community-acquired *versus* healthcare-associated). The proportional hazards assumption was assessed using Schoenfeld residuals, and covariates violating the assumption were incorporated as time-dependent covariates.

All analyses were performed in R (version 4.3.3) using packages *comorbidity*, *survival*, *survminer* and *ggsurvfit*.

### Ethics statement

This study was approved by the ethics committee of the canton of Geneva, Switzerland (CCER: 2024 − 01842).

## Results

Between 1 January 2021 and 31 December 2023, a total of 294 patients with MSSA BSI were included: 57 (19%) CC398 and 237 (81%) non-CC398 (Supplementary Fig. 1). The majority of BSI were healthcare-associated in both groups: 68% (39/57) of CC398 and 62% (146/237) of non-CC398 cases (Table 1). Supplementary Fig. 2 shows the monthly number of included CC398 and non-CC398 cases throughout the study period, stratified by BSI classification.

**Table 1:** Characteristics (A) and clinical outcomes (B) of 294 hospitalised patients with clonal complex 398 (CC398) or non-CC398 methicillin-susceptible *Staphylococcus aureus* identified in at least one blood culture between 1 January 2021 and 31 December 2023

	CC398 MSSA BSI, N= 57 n (%)	Non-CC398 MSSA BSI, N= 237 n (%)	P value
A. Characteristics			
Sex:			0.06
- Male	42 (74)	153 (65)	
- Female	15 (26)	84 (35)	
Age (years):			0.79
- Median	69	70	
- IQR	56-82	56-81	
Charlson comorbidity score			0.39
- Median	3	3	
- IQR	1-5	0-5	
Concurrent SARS-CoV-2 infection	4 (7)	19 (8)	1
Concurrent influenza (A or B) infection	0 (0)	3 (1)	1
Classification of BSI:			0.43
- Community-acquired	18 (32)	91 (38)	
- Healthcare-associated	39 (68)	146 (62)	
B. Clinical outcomes			
Death (all-causes):			
- 7-day	3 (5)	17 (7)	0.83
- 14-day	8 (14)	24 (10)	0.54
- 30-day	9 (16)	34 (14)	0.86
- 90-day	11 (19)	54 (22)	0.79
Admission to ICU	12 (21)	50 (21)	1
Admission to IMCU	22 (39)	98 (41)	0.81
Length of hospital stay after MSSA BSI onset (days):			0.59
- Median	23	24	
- IQR	13-73	12-57	

*BSI* bloodstream infection, *CC398* clonal complex 398, *ICU* intensive care unit, *IMCU* intermediate care unit, *IQR* interquartile range, *MSSA* methicillin-susceptible *Staphylococcus aureus*

Concurrent influenza infection defined as a positive test result within 7 days of MSSA positive blood culture collection. Concurrent SARS-CoV-2 infection defined as a positive test result within 14 days of MSSA positive blood culture collection

**Fig. 1:**
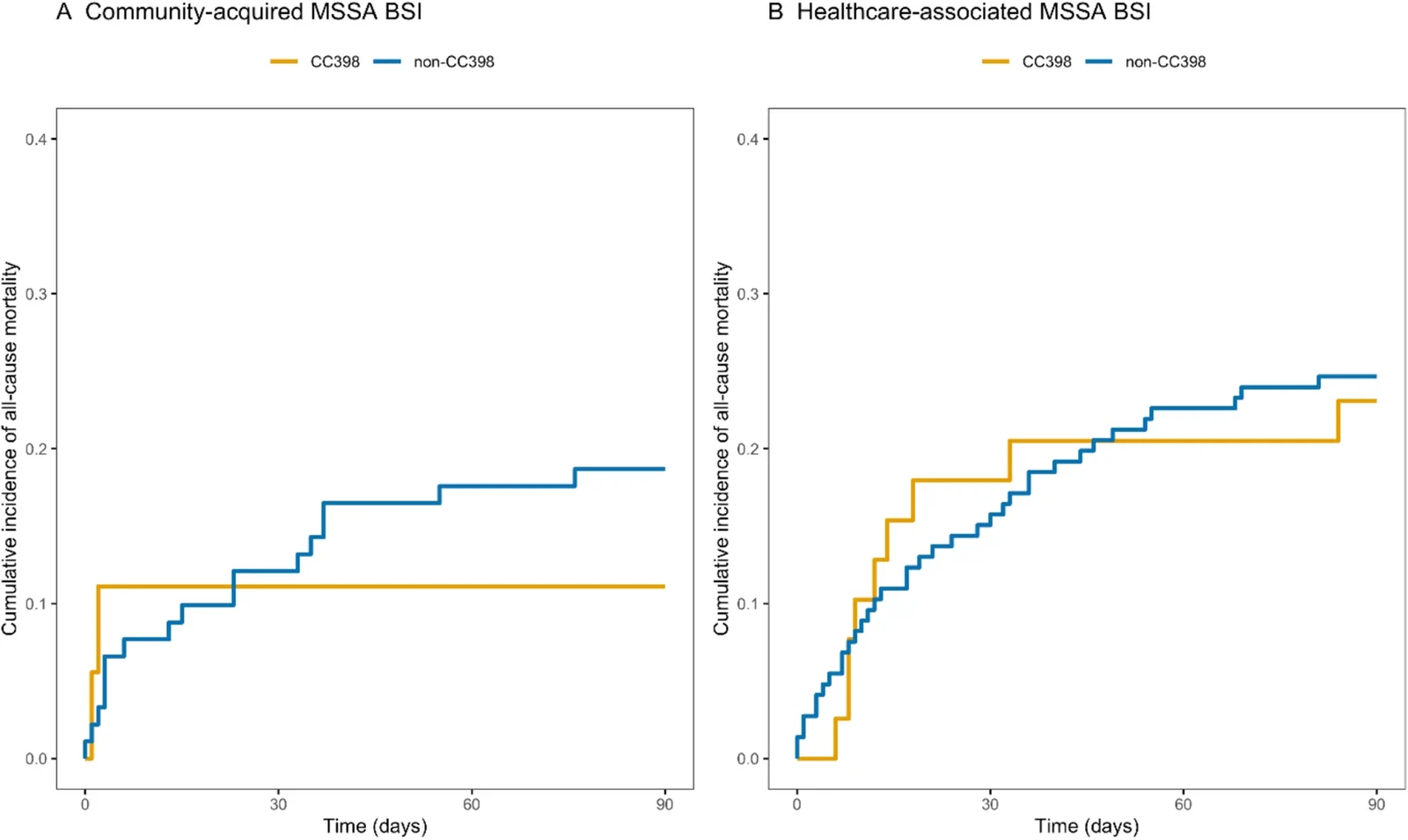
Cumulative incidence of all-cause 90-day mortality following community-acquired (**A**) or healthcare-associated (**B**) methicillin-susceptible *Staphylococcus aureus* bloodstream infection

Crude 90-day mortality was similar in both groups (19% CC398, 22% non-CC398, *p* = 0.79), as was the proportion of patients requiring admission to the ICU (21% in both groups; Table 1). Figure 1 shows the 90-day cumulative incidence of all-cause mortality following MSSA BSI, stratified by site of acquisition and BSI classification. There was no statistically significant difference in 90-day mortality for community-acquired infections (Fig. 1A; weighted log rank test, rho = 1, *p* = 0.5) or healthcare-associated infections (Fig. 1B; weighted log rank test, rho = 1, *p* = 0.9). In adjusted multivariate analyses, neither the adjusted 90-day nor the adjusted 30-day mortality hazard differed significantly between CC398 and non-CC398 MSSA (aHR 90-day mortality 1.17, 95%CI 0.61–2.25, *p* = 0.64; aHR 30-day mortality 0.87, 95%CI 0.41–1.83, *p* = 0.72).

## Discussion

These results indicate no clinically relevant or statistically significant difference in the risk of all-cause mortality between CC398 or non-CC398 MSSA BSI. As such, this retrospective cohort study does not confirm the strong association between CC398 MSSA BSI and increased risk of hospital onset, critical illness, and mortality reported in an earlier study from France [8]. Methodological differences may explain these divergent results: unlike the case-control design of Bouiller et al. [8], our retrospective cohort included all consecutive MSSA BSI cases and assessed 30- and 90-day mortality using time-to-event analyses. Temporal difference may have also contributed, as circulating CC398 subclades may have evolved since the earlier French study conducted between 2010 and 2014. Livestock-independent, human-adapted CC398 lineages, have been increasingly identified among hospitalised patients [12] and may not be inherently more virulent [6]. Genomic studies further support substantial heterogeneity within CC398 [13], suggesting that the clinical impact may be subclade-dependent.

Our findings are strengthened by the assessment of all-cause mortality at 30 and 90 days which considered not only in-hospital deaths, but also deaths recorded in the cantonal registry. We used a conservative definition to classify BSI as healthcare-associated, largely aligned with previous studies [8, 11].

Nevertheless, several limitations must be acknowledged. While cases of MSSA CC398 were identified using PCR-based genotyping, non-CC398 isolates were not further characterized, and virulence factors such as tst, PVL, or erm(T) were not assessed. Unmeasured genetic and phenotypic heterogeneity within both groups may have obscured lineage-specific difference in clinical outcomes. We lacked detailed data on clinical management (including timing and adequacy of source control and antibiotic therapy) and on markers of complicated infection (e.g., infective endocarditis, persistent bacteraemia, deep-seated abscesses, or infection involving implanted prosthetic material). In addition, validated severity scores such as the Pitt bacteraemia score or SOFA score were unavailable, limiting adjustment for acute illness severity and raising the possibility of residual confounding. However, several elements support the robustness of our findings: crude all-cause mortality, ICU/IMCU admission rates, and length of hospital stay were very similar between groups, suggesting broadly comparable overall severity. Moreover, adjusted effect estimates accounting for age, sex, weighted Charlson comorbidity score, and bloodstream infection classification were close to 1 with relatively narrow confidence intervals, indicating no clinically meaningful mortality difference. Notably, our adjusted hazard ratio differed substantially from the effect size reported in the earlier case-control study by Bouiller et al. (aOR 2.44, 95% CI 1.23–4.85), with minimal overlap in confidence intervals compared with our estimate (aHR 0.84, 95% CI 0.41–1.83), making it unlikely that residual confounding alone would explain such a marked discrepancy or uncover a strong detrimental CC398 effect in a more adjusted model. Finally, our time-to-event approach incorporates the temporal dimension of mortality—indirectly capturing aspects of the clinical course—and standards of care at Geneva University Hospitals, including systematic infectious diseases consultation for all *S. aureus* bloodstream infection episodes, likely reduced the probability of major between-group differences in management. Taken together, although residual confounding related to unmeasured markers of infection complexity cannot be entirely excluded, it is unlikely that more granular adjustment would substantially change the clinical or statistical interpretation of our results.

Our findings suggest that while CC398 has become increasingly prevalent among MSSA BSI in Geneva, this has not translated into poorer clinical outcomes. This supports the need for continued molecular and epidemiologic surveillance of MSSA subclades, as well as future multicentre studies to investigate the clinical impact of various MSSA subclades.

## Supplementary Information

Below is the link to the electronic supplementary material.

Supplementary Material 1

## Data Availability

The data that support the findings of this study are not publicly available.
