# Supplementary material for: Mortality of CC398 Staphylococcus aureus bloodstream infections among hospitalised patients in a Swiss university hospital: a retrospective cohort study
Source: Eur J Clin Microbiol Infect Dis. 2026 Apr 7;45(7):2185–9. doi: 10.1007/s10096-026-05493-6 (PMC13652764; doi:10.1007/s10096-026-05493-6)
Supplement: Supplementary file 1 [file 10096_2026_5493_MOESM1_ESM.docx]

**Mortality of CC398 *Staphylococcus aureus* bloodstream infections among hospitalised patients in a Swiss university hospital: a retrospective cohort study**

Davide Bosetti, Rebecca Grant, Etienne Chalot, Mohamed Abbas, Niccolò Buetti, Patrice François, Stephan Harbarth

Corresponding author:

Dr Davide Bosetti

Infection Control Program and WHO Collaborating Centre

Geneva University Hospitals and Faculty of Medicine

Rue Gabrielle-Perret-Gentil 4

Geneva 1205

Switzerland

Email: [davide.bosetti@hug.ch](mailto:davide.bosetti@hug.ch)

**Supplementary Material**

**Supplementary Fig. 1.** Flowchart of included patients with *Staphylococcus aureus* bloodstream infection between 1 January 2021 and 31 December 2023

*Microbiological and molecular characterization*

All isolates included in this analysis were retrieved, subcultured on blood agar plates, and genotyped using a previously described MLVA assay [1] with slight modifications. Briefly, single colonies picked from agar plates and suspended in 200 μl Tris-EDTA buffer (TE; 10 mM Tris and 1 mM EDTA, pH 8) then mixed with 100 mg glass beads (diameter, 100 μm; Sigma-Aldrich, Switzerland) on a vortex agitator at maximum power for 60 sec. After centrifugation at 5,000 rpm for 2 min, the supernatant was directly used in the modified multiplex PCR assay. All PCRs were resolved by using the BioAnalyzer 2100 on a DNA 7,500 chip (Agilent, Palo Alto, CA). Isolates were considered as CC398 by visual inspection of the Bioanalyzer profiles as they harboured the *sau1-hsdS1* gene, a hallmark of the CC398 lineage [2] yielding an amplification product showing an exact size of 300 bp. All other isolates were considered non-CC398 MSSA.

**Supplementary Fig. 2.** Cases of (A) community-acquired (A) and healthcare-associated methicillin-susceptible *Staphylococcus aureus* bloodstream infections (MSSA BSI) per month between 1 January 2021 and 31 December 2023. Each panel shows clonal complex 398 (CC398) or non-CC398 MSSA BSI

**References**

[1] Francois P, Huyghe A, Lew D, Vaudaux P, Pittet D, Schrenzel J. Use of an automated multiple-locus, variable-number tandem repeat-based method for rapid and high-throughput genotyping of Staphylococcus aureus isolates. J Clin Microbiol. 2005 Jul;43(7):3346-55. doi: 10.1128/JCM.43.7.3346-3355.2005.
[2] Stegger M, Lindsay JA, Moodley A, Skov R, Broens EM, Guardabassi L. Rapid PCR detection of Staphylococcus aureus clonal complex 398 by targeting the restriction-modification system carrying sau1-hsdS1. J Clin Microbiol. 2011;49(2):732-734. doi:10.1128/JCM.01970-10.
